# Supplementary material for: Chromothripsis during telomere crisis is independent of NHEJ, and consistent with a replicative origin
Source: Genome Res. 2019 May;29(5):737–49. doi: 10.1101/gr.240705.118 (PMC6499312; doi:10.1101/gr.240705.118)
Supplement: Supplemental Material [file supp_gr.240705.118_Supplemental_file_1.zip › contigs/annotated_contigs/DB57/contig.2.DB57_length_313_mean_cov_11.249201278.docx]

**DB57_length_313_mean_cov_11.249201278**

```
AGAGAGAAACCAGCCTCCTGGATCGGGTGCCTGTGTAGTGACCCCTCCCGAAGGAGGATAAGGTGGTCAGCAGAGGAGATGAGC|TGGT
 >chr17:71731656-71731740 - E=4e-30 p=1e-02
ACACAAAATCAGGATATAATTATTTTTTCAAGTTGGAGTCTCGCTCTGTCACCCAGGCTGGAGTGCAGTGGCACAATCTTCAGCTCACT
 >chr17:66415521-66415746 - E=3e-121
GCAACCTCTGCCTCCCAGGTTCAAGCAATTCTCCTGCCTCAGCCTCCTGAGTAGCTGGGATCACAGGCACCTGCCACCACGCTCGGCTA

ATTTTTGTATTTTTAGTAGAGATGGCGTTTCACCATATTGGCCAGGC
```
